# Examining the predictive accuracy of the novel 3D N-linear algebraic molecular codifications on benchmark datasets

**DOI:** 10.1186/s13321-016-0122-x

**Published:** 2016-02-25

**Authors:** César R. García-Jacas, Ernesto Contreras-Torres, Yovani Marrero-Ponce, Mario Pupo-Meriño, Stephen J. Barigye, Lisset Cabrera-Leyva

**Affiliations:** Escuela de Sistemas y Computación, Pontificia Universidad Católica del Ecuador Sede Esmeraldas (PUCESE), Esmeraldas, Ecuador; Grupo de Investigación de Bioinformática, Centro de Estudio de Matemática Computacional (CEMC), Universidad de las Ciencias Informáticas, La Habana, Cuba; Computer-Aided Molecular “Biosilico” Discovery and Bioinformatics Research International Network (CAMD-BIR IN), Cumbayá, Quito, Ecuador; Instituto de Simulación Computacional (ISC-USFQ), Universidad San Francisco de Quito (USFQ), Diego de Robles y vía Interoceánica, 17-1200-841 Quito, Ecuador; Escuela de Medicina, Colegio de Ciencias de la Salud, Edificio de Especialidades Médicas, Hospital de los Valles, Universidad San Francisco de Quito (USFQ), Av. Interoceánica Km 12 ½ - Cumbayá, Quito, Ecuador; Grupo de Investigación de Inteligencia Artificial (AIRES), Facultad de Informática, Universidad de Camagüey, Camagüey, Cuba; Departamento de Química, Universidade Federal de Lavras, UFLA, Caixa Postal 3037, Lavras, MG 37200-000 Brazil; Departamento de Técnicas de Programación, Facultad 6, Universidad de las Ciencias Informáticas, La Habana, Cuba

**Keywords:** Multiple Linear Regression, QuBiLS-MIDAS, 3D-QSAR, TOMOCOMD-CARDD

## Abstract

**Background:**

Recently, novel 3D alignment-free molecular descriptors (also known as QuBiLS-MIDAS) based on two-linear, three-linear and four-linear algebraic forms have been introduced. These descriptors codify chemical information for relations between two, three and four atoms by using several (dis-)similarity metrics and multi-metrics. Several studies aimed at assessing the quality of these novel descriptors have been performed. However, a deeper analysis of their performance is necessary. Therefore, in the present manuscript an assessment and statistical validation of the performance of these novel descriptors in QSAR studies is performed.

**Results:**

To this end, eight molecular datasets (angiotensin converting enzyme, acetylcholinesterase inhibitors, benzodiazepine receptor, cyclooxygenase-2 inhibitors, dihydrofolate reductase inhibitors, glycogen phosphorylase b, thermolysin inhibitors, thrombin inhibitors) widely used as benchmarks in the evaluation of several procedures are utilized. Three to nine variable QSAR models based on Multiple Linear Regression are built for each chemical dataset according to the original division into training/test sets. Comparisons with respect to *leave*-*one*-*out cross*-*validation correlation coefficients*$\left( {Q_{loo}^{2} } \right)$ reveal that the models based on QuBiLS-MIDAS indices possess superior predictive ability in 7 of the 8 datasets analyzed, outperforming methodologies based on similar or more complex techniques such as: Partial Least Square, Neural Networks, Support Vector Machine and others. On the other hand, superior *external correlation coefficients*$\left( {Q_{ext}^{2} } \right)$ are attained in 6 of the 8 test sets considered, confirming the good predictive power of the obtained models. For the $Q_{ext}^{2}$ values non-parametric statistic tests were performed, which demonstrated that the models based on QuBiLS-MIDAS indices have the best global performance and yield significantly better predictions in 11 of the 12 QSAR procedures used in the comparison. Lastly, a study concerning to the performance of the indices according to several conformer generation methods was performed. This demonstrated that the quality of predictions of the QSAR models based on QuBiLS-MIDAS indices depend on 3D structure generation method considered, although in this preliminary study the results achieved do not present significant statistical differences among them.

**Conclusions:**

As conclusions it can be stated that the QuBiLS-MIDAS indices are suitable for extracting structural information of the molecules and thus, constitute a promissory alternative to build models that contribute to the prediction of pharmacokinetic, pharmacodynamics and toxicological properties on novel compounds.

**Graphical abstract:**
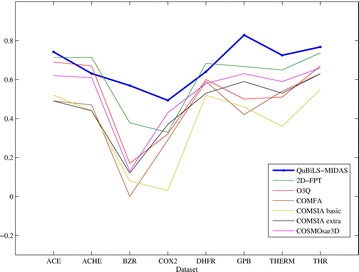
Comparative graphical representation of the performance of the novel QuBiLS-MIDAS 3D-MDs with respect to other methodologies in QSAR modeling of eight chemical datasets

**Electronic supplementary material:**

The online version of this article (doi:10.1186/s13321-016-0122-x) contains supplementary material, which is available to authorized users.

## Background

Computational methods that employ statistical and/or artificial intelligence procedures are widely used in the drug discovery process, where the Quantitative Structure–Activity Relationship (QSAR) studies have an important role [1–4]. These studies are based on the principle that the biological activity (or property) of compounds depends on their structural and physicochemical features and thus, are primarily aimed at finding good correlations among molecular features and specific biological activities [5]. In this way, models with high external predictive ability in novel compounds could be built.

Right from the works developed by Hansch and Fujita in 1960s [6, 7], considered as the origins of the modern QSAR studies [8], several approaches have been reported in the literature with most of these being 2D-QSAR methods, that is, they only consider the topological structural features of molecules often using matrix representations such as the connectivity and distance matrices [8]. However, with the introduction of the CoMFA [9] methodology in 1988, the 3D-QSAR approaches become popular. These take into account the geometric (3D) features of molecules, which can be computed either from the information represented in a *grid* through an alignment process with respect to a reference compound or a pharmacophore [2, 10, 11], or using procedures based on Cartesian coordinates [8, 12, 13], molecular spectra [14, 15] and molecular transforms [16], or by the adaptation of 2D methods to take into account three-dimensional (3D) aspects [17–21].

However, despite the number and variety of procedures defined up to date, there exists continued interest in creating or extending the current approaches to more generalized forms in order to codify more relevant chemical information with the aim of yielding QSAR models with better predictive ability. This assertion is in accordance with the *Non Free Lunch Theorem* [22], which could be interpreted as no single QSAR procedure yields superior predictions than all the others when its performance is averaged over all possible compound datasets. This can be confirmed in a report performed by Sutherland et al. [23], where it is observed how well-established procedures, assessed in eight diverse chemical datasets, present moderate predictions and without significant differences among them (see Additional file 1: Table S1 for a statistical analysis). The justification for this observation is that one family of molecular descriptors (MDs) may not suffice to codify all chemical information and/or molecular properties for different chemical datasets. In other words, the relevance of MDs depends on the nature of the compounds under study. It is therefore necessary to search for alternative methods/approaches to codify novel and orthogonal chemical information.

Inspired by the previous idea, recently the *3D N*-*linear algebraic molecular descriptors* have been introduced as a novel mathematical procedure for computing the structural features of chemical compounds [24–26]. These MDs employ the *bilinear*, *quadratic* and *linear algebraic maps* [27] to codify information between atom-pairs by using several *(dis*-*)similarity metrics* [25]. Also, the *N*-*linear algebraic forms* [28] were used as generalized expressions of the *bilinear*, *quadratic* and *linear algebraic maps*, when relations among three and four atoms are studied [26]. In this way, the geometric matrix [8] was extended to consider for the first time relations for more than two atoms.

Several studies aimed at assessing the quality of this novel descriptor family, also called QuBiLS-MIDAS [*acronym of Quadratic, Bilinear and N*-*Linear Maps based on N*-*tuple Spatial Metric [(Dis)*-*Similarity] Matrices and Atomic Weightings*], were performed and these included an evaluation of the information content (variability) and linear independence using Shannon’s entropy based variability analysis [29] (using IMMAN software [30]) and the principal component analysis (PCA) technique [31], respectively. Also, comparisons with other MDs reported in the literature were performed [25, 26]. In general sense, the results demonstrated that the novel MDs have superior variability than 3D DRAGON indices and another approaches implemented in several software [32–35]. Furthermore, the results revealed that the novel 3D N-linear indices not only do they codify all information contained in the 3D DRAGON MDs, but capture information orthogonal to the latter. Lastly, the QuBiLS-MIDAS MDs were used for modeling the *binding affinity to the corticosteroid*-*binding globulin* (CBG), achieving superior results with respect to other QSAR methodologies (see Tables 8–9 in Ref. [25] and Tables 9–10 in Ref. [26]).

However, although the initial results with QuBiLS-MIDAS MDs are promissory, it cannot be stated that these are most suitable for building QSAR models for all chemical datasets. It is thus necessary to *evaluate the performance of the 3D N*-*linear algebraic MDs in QSAR modeling with different molecular sets*. Therefore, this paper is dedicated to the assessment of the utility of the QuBiLS-MIDAS approach in the prediction of the biological activity in several compound datasets and the comparison of the obtained results with those of other QSAR procedures reported in the literature.

## Mathematical overview of the 3D N-linear algebraic molecular descriptors

In this report, the *total and local*-*fragment 3D N*-*linear Algebraic indices* [25, 26] (also known as QuBiLS-MIDAS) are employed to assess the predictive accuracy of this approach in QSAR studies. These molecular descriptors (MDs) are calculated from the contribution of each atom in a molecule. That is, if a molecule is comprised of *n* atoms then the *kthtwo*-*linear*, *three*-*linear* and *four*-*linear* algebraic indices for each atom “*a*” are computed as N-linear (Multi-linear) algebraic forms (maps) in ${\mathbb{R}}^{n}$, in a canonical basis set, when relations among two (*N* = 2), three (*N* = 3) and four (*N* = 4) atoms are considered, respectively. These descriptors are mathematically expressed as follows:

1 $$\begin{aligned}_{b(F)} L_{a} & = b_{(F)}^{a,k} \left( {\bar{x},\bar{y}} \right) = \mathop \sum \limits_{i = 1}^{n} \mathop \sum \limits_{j = 1}^{n} g_{ij(F)}^{a,k} x^{i} y^{j} = [X]^{T} {\mathbb{G}}_{(F)}^{a,k} [Y] \\ \end{aligned}$$

2 $$\begin{aligned}_{tr(F)} L_{a} & = {tr}_{\left( F \right)}^{a,k} \left( {\bar{x},\bar{y},\bar{z}} \right) = \mathop \sum \limits_{i = 1}^{n} \mathop \sum \limits_{j = 1}^{n} \mathop \sum \limits_{l = 1}^{n} gt_{ijl(F)}^{a,k} x^{i} y^{j} z^{l} = {{\mathbb{G}}{\mathbb{T}}}_{(F)}^{a,k} \cdot \bar{x} \cdot \bar{y} \cdot \bar{z} \\ \end{aligned}$$

3 $$\begin{aligned}_{qu(F)} L_{a} & = qu_{(F)}^{a,k} \left( {\bar{x},\bar{y},\bar{z},\bar{w}} \right) = \mathop \sum \limits_{i = 1}^{n} \mathop \sum \limits_{j = 1}^{n} \mathop \sum \limits_{l = 1}^{n} \mathop \sum \limits_{h = 1}^{n} gq_{ijlh(F)}^{a,k} x^{i} y^{j} z^{l} w^{h} = {{\mathbb{G}}{\mathbb{Q}}}_{(F)}^{a,k} \cdot \bar{x} \cdot \bar{y} \cdot \bar{z} \cdot \bar{w} \\ \end{aligned}$$

where, “*a*” is a specific atom (*a* = 1, 2,…,*n*), *n* is the number of atoms in a molecule, _(*F*)_*L*_*a*_ is the entry corresponding to the contribution of the atom “*a*” in the vector of atom-level indices _(*F*)_*L*, *F* is a local-fragment (group or atom-type) that may or not be considered in the index computation, and *x*^1^,…,*x*^*n*^, *y*^1^,…,*y*^*n*^, *z*^1^,…,*z*^*n*^ and *w*^1^,…,*w*^*n*^ are the values (coordinates or components) of the molecular vectors $\bar{x}$, $\bar{y}$, $\bar{z}$ and $\bar{w}$, respectively. In addition, the coefficients $g_{ij(F)}^{a,k}$, $gt_{ijl(F)}^{a,k}$ and $gq_{ijlh(F)}^{a,k}$ are the elements of the *kthtwo*-*tuple, three*-*tuple* and *four*-*tupleatom*-*level total (or local*-*fragment)spatial*-*(dis)similarity matrices* [${\mathbb{G}}_{\left( F \right)}^{a,k}$, ${{\mathbb{G}}{\mathbb{T}}}_{\left( F \right)}^{a,k}$ and ${{\mathbb{G}}{\mathbb{Q}}}_{\left( F \right)}^{a,k}$], which are obtained from the corresponding *kthtwo*-*tuple, three*-*tuple* and *four*-*tupletotal (or local*-*fragment)spatial*-*(dis)similarity matrices* [${\mathbb{G}}_{\left( F \right)}^{k}$, ${{\mathbb{G}}{\mathbb{T}}}_{\left( F \right)}^{k}$ and ${{\mathbb{G}}{\mathbb{Q}}}_{\left( F \right)}^{k}$]. Lastly, *k* (±1, ±2,…,±12) is the power to which the matrix approaches are raised through the Hadamard product.

The molecular vectors (or property vectors) $\bar{x}$, $\bar{y}$, $\bar{z}$ and $\bar{w}$ are calculated by using the Chemistry Development Kit (CDK) library [36] considering the following fragment- and atom-based properties: atomic mass (m), the van der Waals volume (v), the atomic polarizability (p), atomic electronegativity in Pauling scale (e), atomic Ghose-Crippen LogP (a), Gasteiger-Marsili atomic charge (c), atomic polar surface area (psa), atomic refractivity (r), atomic hardness (h) and atomic softness (s).

The *total matrix approaches*${\mathbb{G}}_{{}}^{k}$, ${{\mathbb{G}}{\mathbb{T}}}_{{}}^{k}$ and ${{\mathbb{G}}{\mathbb{Q}}}_{{}}^{k}$ constitute the basis for the calculation of the *two*-*linear*, *three*-*linear* and *four*-*linear* indices and these are employed to represent the chemical information codified on interactions among “*N*” atoms of a molecule. Specifically, for *k* = 1 (matrix of order 1) the coefficients $g_{ij}^{1}$, $gt_{ijl}^{1}$ and $gq_{ijlh}^{1}$ corresponding to the matrices ${\mathbb{G}}_{{}}^{1}$, ${{\mathbb{G}}{\mathbb{T}}}_{{}}^{1}$ and ${{\mathbb{G}}{\mathbb{Q}}}_{{}}^{1}$ can be calculated by using several *(dis)*-*similarity metrics* and *multi*-*metrics* to capture the information on the relations between two, three and four atoms, respectively [25, 26]. To compute the atom-pair relations, *metrics* (see Table 1) derived from the general Minkowski definition (e.g. Manhattan, Euclidean) as well as others that have been successfully used in machine learning algorithms and similarity/dissimilarity studies (e.g. Canberra, Soergel, Clark) are employed. On the other hand, different *multi*-*metrics* (see Table 2) to calculate the ternary (three) and quaternary (four) relations among atoms of a molecule can be utilized, such as: *bond angle* for relations among three atoms and *dihedral angle* for relations among four atoms. Table 3 shows examples of *two*-*tuple and three*-*tuple total spatial*-*(dis)similarity matrices* calculated with some previously mentioned metrics and multi-metrics.

**Table 1:** Metrics used to compute the “distance” between two atoms of a molecule

Metrics	Formula^a^	Range^b^	Average	Range
Minkowski (**M1–M7**) *p* = 0.25, 0.5, 1, 1.5, 2, 2.5, 3, and ∞ [where, when *p* = 1 it is the Manhattan, city-block or taxi distance (also known as Hamming distance between binary vectors) and *p* = 2 is Euclidean distance)	$d_{XY} = \left( {\mathop \sum \limits_{j = 1}^{h} \left| {x_{j} - y_{j} } \right|^{p} } \right)^{{\frac{1}{p}}}$	[0, ∞)	$\bar{d} = \frac{{d_{XY} }}{{n^{1/p}}}$	[0, ∞)
Chebyshev/Lagrange (**M8**) (Minkowski formula when *p* = ∞)	$d_{XY} = max\left\{ {\left| {x_{j} - y_{j} } \right|} \right\}$			
Canberra (**M10**)	$d_{XY} = \mathop \sum \limits_{j = 1}^{h} \frac{{\left| {x_{j} - y_{j} } \right| }}{{\left| {x_{j} } \right| + \left| {y_{j} } \right|}}$	[0, *n*]	$\bar{d} = \frac{{d_{XY} }}{n}$	[0, 1]
Lance–Williams/Bray–Curtis (**M11**)	$d_{XY} = \frac{{\mathop \sum \nolimits_{j = 1}^{h} \left| {x_{j} - y_{j} } \right| }}{{\mathop \sum \nolimits_{j = 1}^{h} \left( {\left| {x_{j} } \right| + \left| {y_{j} } \right|} \right) }}$	[0, 1]	$\bar{d} = \frac{{d_{XY} }}{n}$	$\left[ {0,\frac{1}{n}} \right]$
Clark/coefficient of divergence (**M12**)	$d_{XY} = \sqrt {\mathop \sum \limits_{j = 1}^{h} \left( {\frac{{x_{j} - y_{j} }}{{\left| {x_{j} } \right| + \left| {y_{j} } \right|}}} \right)^{2} }$	[0, *n*]	$\bar{d} = \frac{{d_{XY} }}{\sqrt n }$	$\left[ {0,\sqrt n } \right]$
Soergel (**M13**)	$d_{XY} = \frac{1}{n}\mathop \sum \limits_{j = 1}^{h} \frac{{\left| {x_{j} - y_{j} } \right| }}{{max\left\{ {x_{j} ,y_{j} } \right\}}}$	[0, 1]	$\bar{d} = \frac{{d_{XY} }}{n}$	$\left[ {0,\frac{1}{n}} \right]$
Bhattacharyya (**M14**)	$d_{XY} = \sqrt {\mathop \sum \limits_{j = 1}^{h} \left( {\sqrt {x_{j} } - \sqrt {y_{j} } } \right)^{2} }$	[0, ∞)	$\bar{d} = \frac{{d_{XY} }}{\sqrt n }$	[0, ∞)
Wave–Edges (**M15**)	$d_{XY} = \mathop \sum \limits_{j = 1}^{h} \left( {1 - \frac{{min\left\{ {x_{j} ,y_{j} } \right\} }}{{max\left\{ {x_{j} ,y_{j} } \right\}}}} \right)$	[0, *n*]	$\bar{d} = \frac{{d_{XY} }}{n}$	[0, 1]
Angular separation/[1 − Cosine (**Ochiai**)] (**M16**)	*d*_*XY*_ = 1−*Cos*_*XY*_ where, $Cos_{XY} = \frac{{\varvec{XY}}}{{\varvec{XY}}} = \frac{{\mathop \sum \nolimits_{j = 1}^{h} x_{j} y_{j} }}{{\sqrt {\mathop \sum \nolimits_{j = 1}^{h} x_{j}^{2} \mathop \sum \nolimits_{j = 1}^{h} y_{j}^{2} } }}$	[0, 2]		

^a^The variables *x*_*j*_ and *y*_*j*_ are the values of the coordinate *j* of the atoms *X* and *Y* of a molecule, respectively. The *h* value is equal to 3 and corresponds to the 3D Cartesian coordinates (x, y, z) of an atom. The *p* values in Minkowski metric are 0.25, 0.5, 1 (Manhattan), 1.5, 2 (Euclidean), 2.5 and 3 (Minkowski)

^b^“*Range*” refers to “range” and not to “rank” and is defined as *Range* = *max*{*x*_*j*_} − *min*{*x*_*j*_}

**Table 2:** Measures used to compute the ternary (A) and quaternary (B) relations (multi-metrics) among atoms of a molecule

Measure	Formula
(A) Ternary measures (*T*_*XYZ*_)	
Perimeter (**M19–M20**)	*T*_*XTZ*_ = *d*_*xy*_ + *d*_*yz*_ + *d*_*zx*_
Triangle area (**M21–M22**)	$$\begin{aligned} T_{XYZ} & = \sqrt {s\left( {s - d_{XY} } \right)\left( {s - d_{YZ} } \right)\left( {s - d_{ZX} } \right)} \\ s & = \frac{{d_{XY} + d_{YZ} + d_{ZX} }}{2} \\ \end{aligned}$$
Sides summation (**M25–M26**)	*T*_*XTZ*_ = *d*_*xy*_ + *d*_*yz*_
Bond angle (angle between sides) (**m27–m28**)	$$\begin{aligned} & A_{X} ,A_{Y} ,A_{Z} \;coordinates\;of\;three\;atoms\;of\;a\;molecule \\ & U = A_{X} - A_{Y} ,\;\;V = A_{Z} - A_{Y} \\ & T_{XYZ} = \alpha = \arccos \left( {\frac{U*V}{\left| U \right|*\left| V \right|}} \right) \\ \end{aligned}$$
(B) Quaternary measures (*T*_*XYZ*_)	
Perimeter (**M19–M20**)	*Q*_*XTZW*_ = *d*_*XY*_ + *d*_*YZ*_ + *d*_*ZW*_ + *d*_*WX*_
Volume (**M23–M24**)	$$\begin{aligned} A_{X} ,A_{Y} ,A_{Z} ,A_{W} \;coordinates\;of\;four\;atoms\;of\;a\;molecule \hfill \\ Q_{XYZW} = \frac{1}{6}\left( {\begin{array}{*{20}c} {A_{Y1} - A_{X1} } & {A_{Z1} - A_{X1} } & {A_{W1} - A_{X1} } \\ {A_{Y2} - A_{X2} } & {A_{Z2} - A_{X2} } & {A_{W2} - A_{X2} } \\ {A_{Y3} - A_{X3} } & {A_{Z3} - A_{X3} } & {A_{W3} - A_{X3} } \\ \end{array} } \right) \hfill \\ \end{aligned}$$
Sides summation (**M25–M26**)	*Q*_*XTZW*_ = *d*_*XY*_ + *d*_*YZ*_ + *d*_*ZW*_
Dihedral angle (**M29–M30**)	$$\begin{aligned} & A_{X} ,A_{Y} ,A_{Z} \;coordinates\;of\;three\;atoms\;of\;a\;molecule\;in\;the\;plane\;A \\ & B_{W} ,B_{Y} ,B_{Z} \;coordinates\;of\;three\;atoms\;of\;a\;molecule\;in\;the\;plane\;B \\ & U_{A} = \left( {A_{X} - A_{Y} } \right) \times \left( {A_{Z} - A_{y} } \right) \\ & U_{B} = \left( {B_{W} - A_{Y} } \right) \times \left( {B_{Z} - A_{y} } \right) \\ & Q_{XYZW} = \alpha = \arccos \left( {\frac{{U_{A} *U_{B} }}{{\left| {U_{A} } \right|*\left| {U_{B} } \right|}}} \right) \\ \end{aligned}$$

**Table 3:** (A) Chemical structure of *Chloro(methoxy)methane* and its labeled molecular scaffold, (B) examples of *two*-*tuple total spatial*-*(dis)similarity matrices* for ***k*** = 1 (order) calculated from different *(dis*-*)similarity metrics*, (C) example of *three*-*tuple total spatial*-*(dis)similarity matrix* for ***k*** = 1 (order) calculated from *bond angle ternary measure*

(A) 3D molecular structure								

(B) Two-tuple total spatial-(dis)similarity matrices, ${\mathbb{G}}^{1}$								
${\mathbb{G}}^{1}$ based on Euclidean metric					${\mathbb{G}}^{1}$ based on Lance-Williams metric			
	C1	C2	O3	Cl4	C1	C2	O3	Cl4
C1	0.000	2.408	1.439	3.939	0.000	1.000	0.973	1.000
C2	2.408	0.000	1.438	1.757	1.000	0.000	0.954	0.293
O3	1.439	1.438	0.000	2.598	0.973	0.954	0.000	0.973
Cl4	3.939	1.757	2.598	0.000	1.000	0.293	0.973	0.000
${\mathbb{G}}^{1}$ based on Soergel metric					${\mathbb{G}}^{1}$ based on Angular Separation metric			
	C1	C2	O3	Cl4	C1	C2	O3	O3
C1	0.000	1.158	1.003	1.709	0.000	1.354	0.558	1.875
C2	1.158	0.000	1.234	1.359	1.354	0.000	0.318	0.237
O3	1.003	1.234	0.000	2.235	0.558	0.318	0.000	0.952
Cl4	1.709	1.359	2.235	0.000	1.875	0.237	0.952	0.000
(C) Three-tuple total spatial-(dis)similarity matrix, ${{\mathbb{G}}{\mathbb{T}}}^{1}$								
${{\mathbb{G}}{\mathbb{T}}}^{1}$ slide 1*ij*					${{\mathbb{G}}{\mathbb{T}}}^{1}$ slide 2*ij*			
	C1	C2	O3	Cl4	C1	C2	O3	O3
C1	0.000	0.000	0.000	0.000	0.000	0.000	0.578	0.281
C2	0.000	0.000	0.578	2.470	0.000	0.000	0.000	0.000
O3	0.000	1.985	0.000	2.682	1.985	0.000	0.000	0.697
Cl4	0.000	0.390	0.163	0.000	0.390	0.000	0.553	0.000
${{\mathbb{G}}{\mathbb{T}}}^{1}$ slide 3*ij*					${{\mathbb{G}}{\mathbb{T}}}^{1}$ slide 4*ij*			
C1	0.000	0.578	0.000	0.297	0.000	0.281	0.297	0.000
C2	0.578	0.000	0.000	1.892	2.470	0.000	1.892	0.000
O3	0.000	0.000	0.000	0.000	2.682	0.697	0.000	0.000
Cl4	0.163	0.553	0.000	0.000	0.000	0.000	0.000	0.000

From these *total matrix approaches* (${\mathbb{G}}^{k}$, ${{\mathbb{G}}{\mathbb{T}}}^{k}$ and ${{\mathbb{G}}{\mathbb{Q}}}^{k}$), local-fragments matrices may be computed in order to consider atom-types or chemical regions of interest and thus yielding the *kthtwo*-*tuple, three*-*tuple and four*-*tuplelocal*-*fragmentspatial*-*(dis)similarity matrices*, denoted by ${\mathbb{G}}_{F}^{k}$, ${{\mathbb{G}}{\mathbb{T}}}_{F}^{k}$ and ${{\mathbb{G}}{\mathbb{Q}}}_{F}^{k}$, respectively (see Eq. 13 in Ref. [25] and Eqs. 17–18 in Ref. [26]). Specifically, the local-fragments (or atom-types), *F*, that could be taken into account to compute these indices include: hydrogen bond acceptors (A), carbon atoms in aliphatic chains (C), hydrogen bond donors (D), halogens (G), terminal methyl groups (M), carbon atoms in aromatic portion (P) and heteroatoms (X) (see Table 4 for examples).

**Table 4:** (A) *Two*-*tuple* ***total*** *spatial*-*(dis)similarity matrix* for ***k*** = 1, ${\mathbb{G}}^{1}$, computed from 3D coordinates of the molecule *Chloro(methoxy)methane* (see Table 1A), (B) examples of *two*-*tuple* ***local-fragment*** *spatial*-*(dis)similarity matrices*, ${\mathbb{G}}_{\varvec{F}}^{1}$, obtained with different chemical fragments

	C1	C2	O3	Cl4
(A) Two-tuple total spatial-(dis)similarity matrices, ${\mathbb{G}}^{1}$				
**C1**	0.000	2.408	1.439	3.939
**C2**	2.408	0.000	1.438	1.757
**O3**	1.439	1.438	0.000	2.598
**Cl4**	3.939	1.757	2.598	0.000
(B) two-tuple local-fragment spatial-(dis)similarity matrices, ${\mathbb{G}}_{F}^{1}$				
${\mathbb{G}}_{F}^{1}$ based on halogens fragment				
**C1**	0.000	0.000	0.000	1.969
**C2**	0.000	0.000	0.000	0.878
**O3**	0.000	0.000	0.000	1.299
**Cl4**	1.969	0.878	1.299	0.000
${\mathbb{G}}_{F}^{1}$ based on methyl groups fragment				
**C1**	0.000	1.204	0.719	1.969
**C2**	1.204	0.000	0.000	0.000
**O3**	0.719	0.000	0.000	0.000
**Cl4**	1.969	0.000	0.000	0.000
${\mathbb{G}}_{F}^{1}$ based on heteroatoms fragment				
**C1**	0.000	0.000	0.719	1.969
**C2**	0.000	0.000	0.719	0.878
**O3**	0.719	0.719	0.000	2.598
**Cl4**	1.969	0.878	2.598	0.000

These *total (or local*-*fragment) matrix approaches* (${\mathbb{G}}_{(F)}^{k}$, ${{\mathbb{G}}{\mathbb{T}}}_{(F)}^{k}$ and ${{\mathbb{G}}{\mathbb{Q}}}_{(F)}^{k}$) are also known as *kthnon*-*stochastictwo*-*tuple, three*-*tuple and four*-*tuple total (or local*-*fragment) spatial*-*(dis)similarity matrices* denoted by $_{ns} {\mathbb{G}}_{(F)}^{k}$, $_{ns} {{\mathbb{G}}{\mathbb{T}}}_{(F)}^{k}$ and $_{ns} {{\mathbb{G}}{\mathbb{Q}}}_{(F)}^{k}$, respectively, because no normalizing procedure is used in their computation. Nonetheless, with the purpose of obtaining normalized matrix representations three probabilistic schemes may be employed to compute the QuBiLS-MIDAS MDs. In this way, the following normalized matrix representations are obtained from the corresponding *non*-*stochastic matrices*: the *kthsimple*-*stochastictwo*-*tuple, three*-*tuple and four*-*tuple total (or local*-*fragment) spatial*-*(dis)similarity matrices* [$_{ss} {\mathbb{G}}_{(F)}^{k}$, $_{ss} {{\mathbb{G}}{\mathbb{T}}}_{(F)}^{k}$ and $_{ss} {{\mathbb{G}}{\mathbb{Q}}}_{(F)}^{k}$] (see Eq. 10 in Ref. [25] and Eqs. 13–14 in Ref. [26]), the *kthdouble*-*stochastictwo*-*tuple total (or local*-*fragment) spatial*-*(dis)similarity matrix* [$_{ds} {\mathbb{G}}_{(F)}^{k}$] (see Sinkhorn–Knopp algorithm in Ref. [37]) and the *kthmutual probabilitytwo*-*tuple, three*-*tuple and four*-*tuple total (or local*-*fragment) spatial*-*(dis)similarity matrices* [$_{mp} {\mathbb{G}}_{(F)}^{k}$, $_{mp} {{\mathbb{G}}{\mathbb{T}}}_{(F)}^{k}$ and $_{mp} {{\mathbb{G}}{\mathbb{Q}}}_{(F)}^{k}$] (see Eq. 12 in Ref. [25] and Eqs. 15–16 in Ref. [26]). Table 5 shows the results obtained with the three probabilistic transformations on a *two*-*tuple total spatial*-*(dis)similarity matrix*.

**Table 5:** Example of probabilistic transformations on the *non*-*stochastic two*-*tuple total spatial*-*(dis)similarity matrix* for ***k*** = 1, $_{{\varvec{ns}}} {\mathbb{G}}^{1}$, computed from 3D coordinates of the *Chloro(methoxy)methane* compound (see Table 1A) by using the Euclidean metric

	**C1**	**C2**	**O3**	**Cl4**	**C1**	**C2**	**O3**	**Cl4**
	Non-stochastic matrix, $_{ns} {\mathbb{G}}^{1}$				Simple-stochastic matrix, $_{ss} {\mathbb{G}}^{1}$			
**C1**	0.000	2.408	1.439	3.939	0.000	0.309	0.185	0.506
**C2**	2.408	0.000	1.438	1.757	0.430	0.000	0.257	0.314
**O3**	1.439	1.438	0.000	2.598	0.263	0.263	0.000	0.475
**Cl4**	3.939	1.757	2.598	0.000	0.475	0.212	0.313	0.000
	Double-stochastic matrix, $_{ds} {\mathbb{G}}^{1}$				Mutual probability matrix, $_{mp} {\mathbb{G}}^{1}$			
**C1**	0.000	0.387	0.246	0.368	0.000	0.089	0.053	0.145
**C2**	0.387	0.000	0.368	0.246	0.089	0.000	0.053	0.065
**O3**	0.246	0.368	0.000	0.387	0.053	0.053	0.000	0.096
**Cl4**	0.368	0.246	0.387	0.000	0.145	0.065	0.096	0.000

**Scheme 1:**
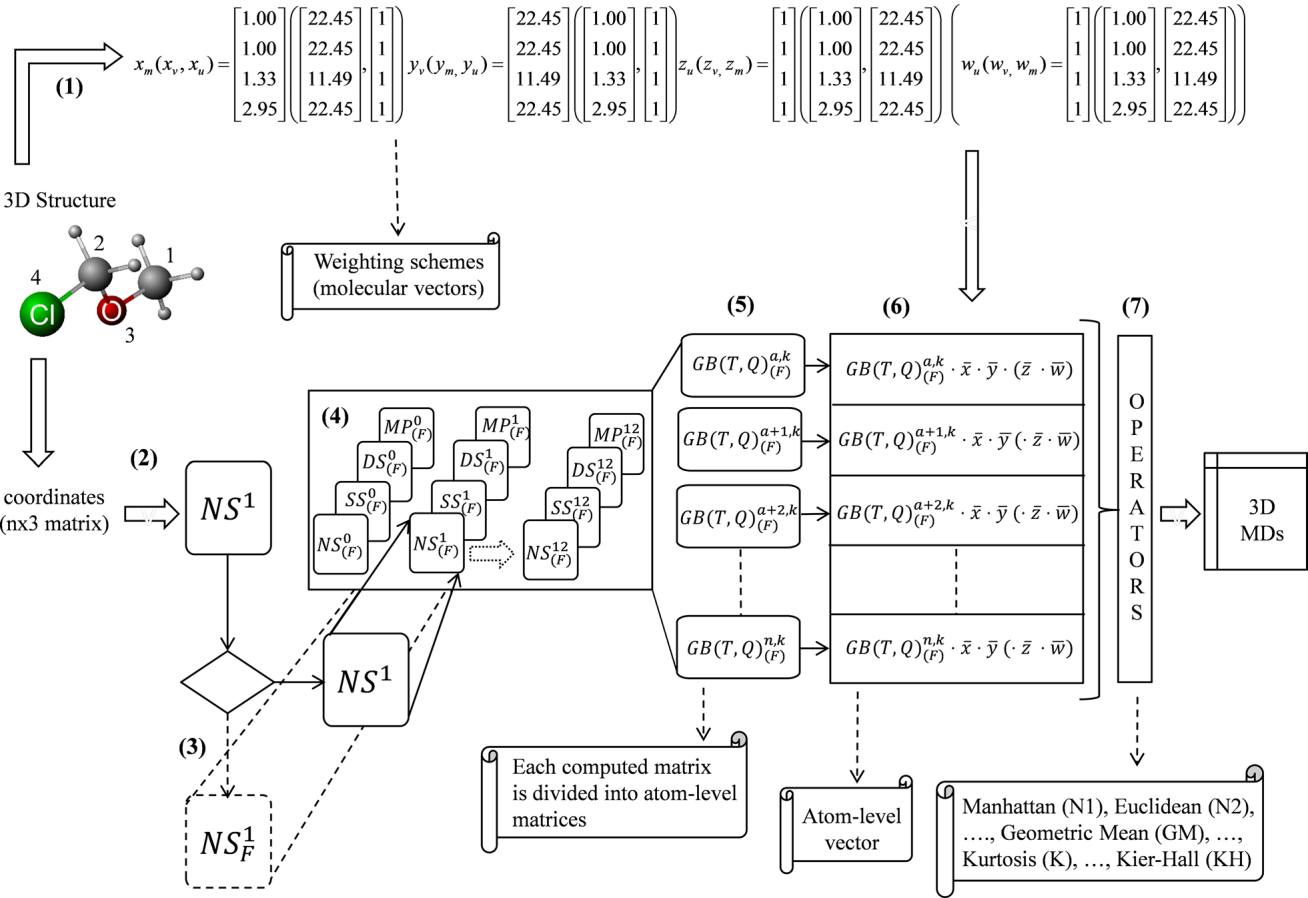
General workflow for calculating the QuBiLS MIDAS molecular descriptors. **(1)** Computation of the molecular vectors according to selected atomic properties; **(2)** Computation from 3D Cartesian coordinates of each atom of a molecule the *non*-*stochastic two*-*tuple, three*-*tuple* or *four*-*tuple* *total spatial*-*(dis)similarity matrices* for ***k*** = 1; **(3)** Consideration of atom-types or local-fragments (optional); **(4)** Computation of the *simple*-*stochastic*, *double*-*stochastic* and *mutual probability* matrices, as well as to determine the ***k***th matrices through Hadamard product until the *k* value selected; **(5)** Splitting the calculated matrices into atom-level matrices; **(6)** Computation of the atom-level indices (descriptors) using the molecular vectors calculated in the step **(1)**; and **(7)** Application of the selected aggregation operators over vector of atom-level descriptors

**Scheme 2:**
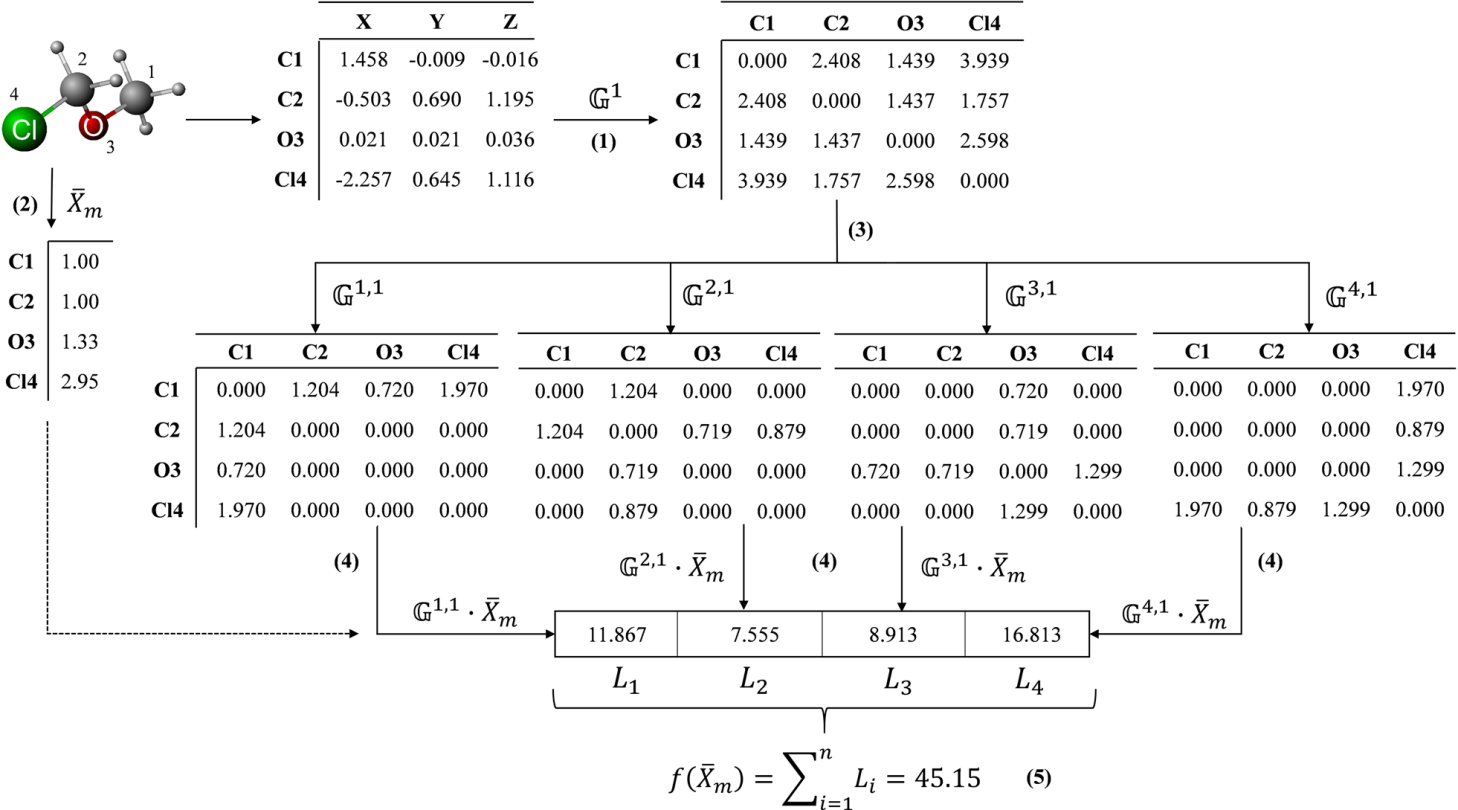
General workflow for the calculation of a *two*-*linear descriptor* based on the *linear algebraic form*, *Euclidean metric*, *non*-*stochastic matrix approach*, *atomic mass as property* and *Manhattan aggregation operator*. **(1)** Computation of the non-stochastic matrix for ***k*** = 1 $\left( {{\mathbb{G}}^{1} } \right)$ from the 3D coordinates matrix and using the Euclidean metric; **(2)** Computation of the molecular vector based on the atomic mass property, $\bar{\varvec{X}}_{\varvec{m}}$; **(3)** Splitting of the ${\mathbb{G}}^{1}$ matrix into “*n*” (number of atoms) atom-level matrices, ${\mathbb{G}}^{{\varvec{a},1}}$, where “*a*” is an atom of the molecule; **(4)** Computation of the atom-level descriptors and saving them into vector ***L***; and **(5)** Application of the Manhattan aggregation operator over the entries of the vector ***L***, being this value the molecular descriptor

Finally, from the *non*-*stochastic (simple*-*stochastic, double*-*stochastic or mutual*-*probability) total (or local*-*fragment) matrices* [i.e. ${\mathbb{G}}_{(F)}^{k}$, ${{\mathbb{G}}{\mathbb{T}}}_{(F)}^{k}$ and ${{\mathbb{G}}{\mathbb{Q}}}_{(F)}^{k}$], the corresponding *atom*-*level matrices* [denoted as ${\mathbb{G}}_{\left( F \right)}^{a,k}$, ${{\mathbb{G}}{\mathbb{T}}}_{\left( F \right)}^{a,k}$ and ${{\mathbb{G}}{\mathbb{Q}}}_{\left( F \right)}^{a,k}$, respectively] are calculated and their coefficients are used in the descriptors calculation (see Eqs. 1–3). Each *atom*-*level matrix* determines an *atom*-*level index* for atom “*a*” of a molecule and this value constitutes a component (entry) of the vector _(*F*)_*L*. Once the vector _(*F*)_*L* is computed then the global definition of the *kthtwo*-*linear*, *three*-*linear* and *four*-*linear* algebraic indices is obtained by applying over the entries of _(*F*)_*L* one or several aggregation operators (see Additional file 1: Table S2 for mathematical definition) [25, 26], which have been successfully employed in other reports [38–40]. In the Scheme 1 a general flowchart regarding the calculation process of the QuBiLS MIDAS MDs detailed in this section may be observed, while Scheme 2 is a graphic representation of each step employed in the computation of a specific *two*-*linear algebraic index*.

In order to automatize the calculation of the 3D N-linear algebraic indices used in the present manuscript the QuBiLS-MIDAS software has been developed [41]. This software has as one of its main features the multi-core processing of the MDs, as well as the option to carry out the distributed calculation of the indices by using the Multi-Server Distributed Computing Platform known as T-arenal [42]. The latter is particularly useful for high-throughput calculation tasks. Both software are freely available via internet at: http://tomocomd.com/.

## Methods

In order to assess the correlation ability of the QuBiLS-MIDAS MDs for different biological activities eight well-known chemical datasets were used. These were previously employed by Sutherland et al. in a comparative study of QSAR methods commonly used in chemo-informatics analysis [23] and since then, these have been utilized as “benchmarks” for comparing results obtained in other approaches [43–47]. These datasets are comprised by angiotensin converting enzyme (ACE) inhibitors, acetylcholinesterase (AchE) inhibitors, ligands for the benzodiazepine receptor (BZR), cyclooxygenase-2 (COX2) inhibitors, dihydrofolate reductase inhibitors (DHFR), inhibitors of glycogen phosphorylase b (GPB), thermolysin inhibitors (THER) and thrombin inhibitors (THR). In this study the 3D coordinates were generated using CORINA software, and the same partitioning into training and test sets used in the initial study was considered in order to guarantee comparability of results.

For these datasets, several configurations based on 3D *two*-*linear*, *three*-*linear* and *four*-*linear algebraic indices* were computed (see Additional file 1: Table S3) using the QuBiLS MIDAS software [41]. Due to the fact that numerous MDs are computed with this program yielding a high-dimensional space, then strategies for data reduction are necessary. In this sense, the following workflow for each set of indices calculated for each chemical dataset was performed only considering the training set compounds:

- The 1000 MDs with best variability behavior according to their Shannon’s Entropy values [29] were retained by using the IMMAN software [30].
- The MDs with values represented as power of 10 (scientific notation) and whose exponents are greater or lesser than ±5 were removed.
- Filters for removing the MDs with correlation equal or greater than 0.95 and standardized entropy lesser than 0.3 were applied.
- The statistical method Multiple Linear Regression (MLR) implemented in the STATISTICA software was employed in order to select the MDs included in the model by using *Forward Stepwise* and *Backward Stepwise* selection procedures.
- The MDs retained after applying the previous steps and computed for the same compounds were merged into a single dataset.

With the reduced data matrices for each chemical datasets, QSAR models were built with the MLR technique to determine the relationship between the response (activity) and predictor variables (MDs). The MLR technique is coupled with the Genetic Algorithm (GA) meta-heuristic as the variable selection method [48]. This strategy (MLR + GA) is implemented in the MobyDigs software (version 1.0) which was utilized to carry out this study [49]. In this sense, to perform the search process several populations with 100 3D N-linear MDs each were created, while the following configurations were used for the GA procedure: *Number of iterations* equal to 500,000, *Population size* equal to 100, *Reproduction/mutation trade*-*off* equal to 0.5, *Selection bias* was initially set to 0 (indicative of random selection) until achieving the 80 % of the maximum number of iterations and was later set to 1 (indicates tournament selection) in order to increase the selection pressure. The values of the previous parameters were selected according to the study performed by Todeschini et al. in Ref. [49].

The search process was carried out by using the $Q_{loo}^{2}$ (“leave-one-out” cross validation) statistical parameter as the fitness function. Once the exploration in each population was completed, then the MDs included in the built 9-variable models were retained with the purpose of creating new populations until 100 MDs. This process is repeated until achieving an only one population with 100 MDs as maximum. Finally, from the final population and for each compound dataset, 3–9 variable regression models were built for the corresponding biological activity. However, as the MobyDigs software generates a set of MLR models then the choice of the most suitable model was performed according to the following steps:

- The “best” 50 models according to the $Q_{loo}^{2}$ parameter were retained.
- To each model retained the validation methods “*bootstrapping*” [50] $\left( {Q_{boot}^{2} } \right)$ and “*Y*-*scrambling*” [51] (*a*(*Q*^2^)) were applied in order to assess the predictive power and the possible chance correlation with respect to the modeled biological activity, respectively. The former randomly creates training sets (with repeated objects) of the same size as the original and the objects left out constitute the test set, while the latter randomly changes the true response variables to determine the quality of the model. Both procedures were repeated 5000 and 300 times, respectively. These methods were applied due to the fact that $Q_{loo}^{2}$ procedure does not suffice to validate the stability of a predictive model [52].
- For each model the function $f(x) = \left( {1 - Q_{boot}^{2} } \right) + \left| {a\left( {Q^{2} } \right)} \right|$ was computed, which takes into account the results obtained with the two validation procedures employed and the model with the smallest *f*(*x*) value constitutes the “best” regression model.
- The “best” regression model was assessed by using “*external validation*” $\left( {Q_{ext}^{2} } \right)$ procedure in the corresponding test set in order to measure its *generalization ability*.

## Results and discussion

### Assessment of the QuBiLS-MIDAS models versus other approaches

In this section the performance of the QuBiLS-MIDAS models for the chemical datasets described in section “Methods” is compared with respect to 16 QSAR methodologies (or descriptor sets) reported in the literature. The Table 6 shows the statistical parameters and equations of the best regression model based on *total and local*-*fragment QuBiLS*-*MIDAS indices* corresponding to each chemical dataset used in this report. In general sense, it can be observed that the bootstrapping validation coefficient $\left( {Q_{boot}^{2} } \right)$ calculated for each model presents a value greater than 0.6, indicative of the good predictive power of the built models. Also, the coefficients computed from scrambling tests (*a*(*Q*^2^)) have in all cases values inferior to 0.4, indicating reduced propensity to chance correlation. Lastly, the values achieved in the external prediction $\left( {Q_{ext}^{2} } \right)$ suggest that the models based on QuBiLS-MIDAS MDs have appropriate generalization ability, given that all $Q_{ext}^{2}$ parameters present values superior to 49 % of the total variance even when outlier compounds are retained in the validation set.

**Table 6:** Statistical parameters and equations of the best models developed for each chemical dataset analyzed

Size	*R* ^2^	$\left( {Q_{\text{loo}}^{2} } \right)$	$\left( {Q_{\text{boot}}^{2} } \right)$	a(*Q* ^2^)	$\left( {Q_{\text{ext}}^{2} } \right)$	SDEP_ext_	Models^a^
ACE dataset							
6	0.814	0.7756	0.765	−0.169	0.7422	1.078	**Act** **=** 1.576 (±1.283) + 0.132 (±0.018) ${}_{{\varvec{NS}2}}^{{\varvec{SD}}} \varvec{TrC}_{\varvec{e}}^{{\varvec{M}20\left( {\varvec{M}4} \right)}}$ − 17.977 (±3.649) ${}_{{\varvec{SS}2}}^{{\varvec{RA}}} \varvec{B}_{{\varvec{a} - \varvec{c}}}^{{\varvec{M}1}}$ + 2.135 (±0.398) ${}_{{\varvec{SS}0}}^{{\varvec{RA}}} \varvec{B}_{{\varvec{a} - \varvec{e}}}^{{}}$ − 3.900 (±0.772) ${}_{{\varvec{SS}1}}^{{\varvec{RA}}} \varvec{F}_{\varvec{a}}^{{\varvec{M}1}}$ + 0.034 (±0.013) $\left[ {{}_{{\varvec{NS}3}}^{{\varvec{AC}\left[ 3 \right]\_\varvec{K}}} \varvec{TrC}_{\varvec{c}}^{{\varvec{M}20\left( {\varvec{M}16} \right)}} } \right]^{D}$ − 0.114 (±0.071) $\left[ {{}_{{\varvec{MP}1}}^{{\varvec{RA}}} \varvec{QuQd}_{\varvec{e}}^{{\varvec{M}29}} } \right]^{\varvec{X}}$
ACHE dataset							
8	0.738	0.6574	0.626	−0.213	0.6309	0.784	**Act** **=** 7.622 (±0.564) − 0.010 (±0.004) ${}_{{\varvec{SS}4}}^{{\varvec{i}50}} \varvec{TrQB}_{{\varvec{e} - \varvec{v}}}^{{\varvec{M}21\left( {\varvec{M}3} \right)}}$ − 0.204 (±0.046) ${}_{{\varvec{NS}4}}^{\varvec{K}} \varvec{Tr}_{{\varvec{a} - \varvec{e} - \varvec{h}}}^{{\varvec{M}21\left( {\varvec{M}1} \right)}}$ + 3.311 (±0.673) ${}_{{\varvec{SS}1}}^{{\varvec{i}50}} \varvec{B}_{{\varvec{a} - \varvec{h}}}^{{\varvec{M}1}}$ − 111.324 (±30.793) ${}_{{\varvec{MP}2}}^{{\varvec{i}50}} \varvec{F}_{\varvec{a}}^{{\varvec{M}1}}$ − 0.413 (±0.156) ${}_{{\varvec{SS}7}}^{{\varvec{ES}\_\varvec{SD}}} \varvec{TrB}_{{\varvec{a} - \varvec{e}}}^{{\varvec{M}21\left( {\varvec{M}13} \right)}}$ − 0.647 (±0.201) ${}_{{\varvec{NS}4}}^{{\varvec{TS}\left[ 2 \right]\_\varvec{K}}} \varvec{B}_{{\varvec{a} - \varvec{v}}}^{{\varvec{M}4}}$ + 0.022 (±0.011) $\left[ {{}_{{\varvec{NS}4}}^{\varvec{K}} \varvec{Tr}_{{\varvec{a} - \varvec{e} - \varvec{h}}}^{{\varvec{M}21\left( {\varvec{M}1} \right)}} } \right]^{\varvec{A}}$ − 1.747 (±0.699) $\left[ {{}_{{\varvec{SS}1}}^{{\varvec{i}50}} \varvec{B}_{{\varvec{a} - \varvec{h}}}^{{\varvec{M}1}} } \right]^{\varvec{P}}$
BZR dataset							
9	0.754	0.6931	0.669	−0.170	0.5692	0.631	**Act** **=** 8.589 (±0.592) + 0.160 (±0.024) ${}_{{\varvec{SS}7}}^{{\varvec{TS}\left[ 4 \right]\_\varvec{K}}} \varvec{Tr}_{{\varvec{a} - \varvec{e} - \varvec{h}}}^{{\varvec{M}19\left( {\varvec{M}11} \right)}}$ + 0.416 (±0.076) ${}_{{\varvec{SS}1}}^{{\varvec{RA}}} \varvec{B}_{{\varvec{c} - \varvec{v}}}^{{\varvec{M}2}}$ + 0.018 (±0.006) ${}_{{\varvec{SS}2}}^{{\varvec{i}50}} \varvec{TrB}_{{\varvec{e} - \varvec{v}}}^{{\varvec{M}19\left( {\varvec{M}16} \right)}}$ + 0.092 (±0.034) ${}_{{\varvec{NS}2}}^{{\varvec{TS}\left[ 7 \right]\_\varvec{K}}} \varvec{Tr}_{{\varvec{a} - \varvec{h} - \varvec{c}}}^{{\varvec{M}27}}$ + 0.030 (±0.010) ${}_{{\varvec{NS}2}}^{{\varvec{AC}\left[ 1 \right]\_\varvec{K}}} \varvec{B}_{{\varvec{c} - \varvec{e}}}^{{\varvec{M}2}}$ − 7.940 (±2.981) ${}_{{\varvec{SS}0}}^{{\varvec{TS}\left[ 4 \right]\_\varvec{i}50}} \varvec{B}_{{\varvec{a} - \varvec{c}}}^{{}}$ − 0.009 (±0.005) $\left[ {{}_{{\varvec{SS}4}}^{{\varvec{AC}\left[ 4 \right]\_\varvec{K}}} \varvec{TrB}_{{\varvec{e} - \varvec{v}}}^{{\varvec{M}20\left( {\varvec{M}13} \right)}} } \right]^{D}$ + 0. (±0.) $\left[ {{}_{{\varvec{NS}4}}^{{\varvec{AM}}} \varvec{QuQd}_{\varvec{v}}^{{\varvec{M}26\left( {\varvec{M}8} \right)}} } \right]^{C}$ + 0. (±0.) $\left[ {{}_{{\varvec{NS}4}}^{{\varvec{AM}}} \varvec{QuQd}_{\varvec{v}}^{{\varvec{M}26\left( {\varvec{M}8} \right)}} } \right]^{P}$
COX2 dataset							
9	0.670	0.6313	0.615	−0.091	0.4932	1.038	**Act** **=** –94.390 (±8.607) + 1.759 (±0.150) ${}_{{\varvec{MP}3}}^{{\varvec{ES}\_\varvec{N}1}} \varvec{B}_{{\varvec{v} - \varvec{e}}}^{{\varvec{M}3}}$ − 0.032 (±0.007) ${}_{{\varvec{NS}4}}^{{\varvec{AC}\left[ 1 \right]\_\varvec{K}}} \varvec{B}_{{\varvec{a} - \varvec{e}}}^{{\varvec{M}13}}$ + 0.317 (±0.070) ${}_{{\varvec{SS}0}}^{{\varvec{ES}\_\varvec{i}50}} \varvec{B}_{{\varvec{h} - \varvec{e}}}$ + 0.005 (±0.002) ${}_{{\varvec{SS}2}}^{{\varvec{SD}}} \varvec{TrQB}_{{\varvec{v} - \varvec{h}}}^{{\varvec{M}20\left( {\varvec{M}16} \right)}}$ + 0.021 (±0.005) ${}_{{\varvec{NS}4}}^{{\varvec{TS}\left[ 5 \right]\_\varvec{K}}} \varvec{B}_{{\varvec{a} - \varvec{c}}}^{{\varvec{M}11}}$ + 0.081 (±0.017) ${}_{{\varvec{NS}2}}^{{\varvec{AC}\left[ 1 \right]\_\varvec{K}}} \varvec{B}_{{\varvec{c} - \varvec{e}}}^{{\varvec{M}8}}$ − 17.442 (±3.695) $\left[ {{}_{{\varvec{SS}4}}^{{\varvec{SD}}} \varvec{QuCB}_{{\varvec{h} - \varvec{c}}}^{{\varvec{M}26\left( {\varvec{M}8} \right)}} } \right]^{\varvec{D}}$ − 14.761 (±2.510) $\left[ {{}_{{\varvec{SS}4}}^{{\varvec{SD}}} \varvec{QuCB}_{{\varvec{h} - \varvec{c}}}^{{\varvec{M}26\left( {\varvec{M}8} \right)}} } \right]^{\varvec{M}}$ + 122.311 (±50.893) $\left[ {{}_{{\varvec{MP}1}}^{{\varvec{SD}}} \varvec{Tr}_{{\varvec{a} - \varvec{h} - \varvec{c}}}^{{\varvec{M}20\left( {\varvec{M}16} \right)}} } \right]^{X}$
DHFR dataset							
9	0.732	0.7055	0.697	−0.077	0.6405	0.826	**Act** **=** 3.127 (±0.519) + 0.019 (±0.005) ${}_{{\varvec{SS}1}}^{{\varvec{RA}}} \varvec{TrB}_{{\varvec{e} - \varvec{v}}}^{{\varvec{M}21\left( {\varvec{M}2} \right)}}$ + 0.050 (±0.007) ${}_{{\varvec{NS}6}}^{{\varvec{GV}\left[ 4 \right]\_\varvec{K}}} \varvec{B}_{{\varvec{c} - \varvec{e}}}^{{\varvec{M}4}}$ − 15.592 (±3.530) ${}_{{\varvec{MP}4}}^{{\varvec{TS}\left[ 2 \right]\_\varvec{i}50}} \varvec{QuQd}_{\varvec{m}}^{{\varvec{M}25\left( {\varvec{M}3} \right)}}$ − 0.067 (±0.007) ${}_{{\varvec{NS}2}}^{{\varvec{GV}\left[ 3 \right]\_\varvec{K}}} \varvec{B}_{{\varvec{a} - \varvec{c}}}^{{\varvec{M}1}}$ + 0.471 (±0.034) ${}_{{\varvec{NS}3}}^{{\varvec{GV}\left[ 1 \right]\_\varvec{K}}} \varvec{B}_{{\varvec{h} - \varvec{c}}}^{{\varvec{M}3}}$ − 0.325 (±0.037) ${}_{{\varvec{NS}1}}^{{\varvec{TS}\left[ 4 \right]\_\varvec{N}1}} \varvec{B}_{{\varvec{c} - \varvec{e}}}^{{\varvec{M}1}}$ + 55.107 (±10.603) ${}_{{\varvec{NS}1}}^{{\varvec{GV}\left[ 5 \right]\_\varvec{SD}}} \varvec{B}_{{\varvec{c} - \varvec{e}}}^{{\varvec{M}3}}$ + 0.044 (±0.008) ${}_{{\varvec{NS}2}}^{{\varvec{TS}\left[ 3 \right]\_\varvec{SD}}} \varvec{B}_{{\varvec{v} - \varvec{e}}}^{{\varvec{M}4}}$ − 0.933 (±0.331) ${}_{{\varvec{MP}4}}^{{\varvec{N}1}} \varvec{Qu}_{{\varvec{e} - \varvec{v} - \varvec{h} - \varvec{c}}}^{{\varvec{M}26\left( {\varvec{M}3} \right)}}$
GPB dataset							
8	0.893	0.8124	0.774	−0.394	0.8283	0.499	**Act** **=** 2.073 (±0.351) + 0.334 (±0.078) ${}_{{\varvec{SS}3}}^{{\varvec{TS}\left[ 4 \right]\_\varvec{K}}} \varvec{TrB}_{{\varvec{e} - \varvec{h}}}^{{\varvec{M}20\left( {\varvec{M}8} \right)}}$ + 0.147 (±0.051) ${}_{{\varvec{NS}2}}^{{\varvec{AC}\left[ 3 \right]\_\varvec{K}}} \varvec{F}_{\varvec{e}}^{{\varvec{M}8}}$ + 0.046 (±0.009) ${}_{{\varvec{SS}3}}^{{\varvec{AC}\left[ 4 \right]\_\varvec{N}1}} \varvec{B}_{{\varvec{c} - \varvec{v}}}^{{\varvec{M}12}}$ + 55.958 (±10.078) ${}_{{\varvec{SS}2}}^{{\varvec{AC}\left[ 2 \right]\_\varvec{N}1}} \varvec{B}_{{\varvec{a} - \varvec{c}}}^{{\varvec{M}8}}$ + 0.050 (±0.039) ${}_{{\varvec{SS}4}}^{{\varvec{N}1}} \varvec{Tr}_{{\varvec{e} - \varvec{v} - \varvec{c}}}^{{\varvec{M}19\left( {\varvec{M}12} \right)}}$ + 0.078 (±0.055) ${}_{{\varvec{NS}3}}^{{\varvec{GV}\left[ 2 \right]\_\varvec{K}}} \varvec{F}_{\varvec{a}}^{{\varvec{M}11}}$ + 1.322 (±0.427) ${}_{{\varvec{MP}0}}^{{\varvec{SD}}} \varvec{QuQTr}_{{\varvec{e} - \varvec{v} - \varvec{h}}}^{{}}$ − 0.309 (±0.108) ${}_{{\varvec{MP}4}}^{{\varvec{SD}}} \varvec{QuQTr}_{{\varvec{e} - \varvec{v} - \varvec{h}}}^{{\varvec{M}26\left( {\varvec{M}3} \right)}}$
THER dataset							
7	0.815	0.7530	0.723	−0.260	0.7248	1.197	**Act** **=** –11.296 (±3.486) + 126.508 (±41.628) ${}_{{\varvec{NS}1}}^{{\varvec{GV}\left[ 5 \right]\_\varvec{N}1}} \varvec{B}_{{\varvec{a} - \varvec{c}}}^{{\varvec{M}8}}$ + 0.016 (±0.003) ${}_{{\varvec{NS}1}}^{{\varvec{GV}\left[ 7 \right]\_\varvec{i}50}} \varvec{Q}_{\varvec{e}}^{{\varvec{M}8}}$ − 4.265 (±0.851) ${}_{{\varvec{SS}1}}^{{\varvec{N}1}} \varvec{Tr}_{{\varvec{v} - \varvec{h} - \varvec{c}}}^{{\varvec{M}20\left( {\varvec{M}3} \right)}}$ + 0.718 (±0.171) ${}_{{\varvec{SS}3}}^{{\varvec{RA}}} \varvec{TrC}_{\varvec{e}}^{{\varvec{M}20\left( {\varvec{M}3} \right)}}$ + 0.016 (±0.009) ${}_{{\varvec{SS}4}}^{{\varvec{RA}}} \varvec{TrB}_{{\varvec{e} - \varvec{v}}}^{{\varvec{M}27}}$ − 0.027 (±0.029) $\left[ {{}_{{\varvec{SS}4}}^{{\varvec{RA}}} \varvec{TrB}_{{\varvec{e} - \varvec{v}}}^{{\varvec{M}27}} } \right]^{A}$ + 0.042 (±0.027) $\left[ {{}_{{\varvec{SS}4}}^{{\varvec{RA}}} \varvec{TrB}_{{\varvec{e} - \varvec{v}}}^{{\varvec{M}27}} } \right]^{X}$
THR dataset							
9	0.866	0.8149	0.789	−0.286	0.7674	0.540	**Act** **=** 5.251 (±0.605) − 2120.900 (±253.086) ${}_{{\varvec{MP}2}}^{{\varvec{TS}\left[ 1 \right]\_\varvec{i}50}} \varvec{Tr}_{{\varvec{a} - \varvec{h} - \varvec{c}}}^{{\varvec{M}19\left( {\varvec{M}2} \right)}}$ − 0.0001 (±0.) ${}_{{\varvec{NS}0}}^{{\varvec{TS}\left[ 5 \right]\_\varvec{i}50}} \varvec{Tr}_{{\varvec{e} - \varvec{v} - \varvec{h}}}^{{}}$ + 0.060 (±0.013) ${}_{{\varvec{SS}1}}^{{\varvec{AC}\left[ 2 \right]\_\varvec{K}}} \varvec{TrQB}_{{\varvec{a} - \varvec{c}}}^{{\varvec{M}27}}$ + 0.022 (±0.004) ${}_{{\varvec{NS}3}}^{{\varvec{RA}}} \varvec{Tr}_{{\varvec{e} - \varvec{v} - \varvec{h}}}^{{\varvec{M}20\left( {\varvec{M}2} \right)}}$ + 1.415 (±0.222) ${}_{{\varvec{NS}2}}^{{\varvec{RA}}} \varvec{TrQB}_{{\varvec{a} - \varvec{c}}}^{{\varvec{M}20\left( {\varvec{M}8} \right)}}$ + 0.958 (±0.293) ${}_{{\varvec{NS}2}}^{{\varvec{GV}\left[ 4 \right]\_\varvec{PN}}} \varvec{B}_{{\varvec{c} - \varvec{v}}}^{{\varvec{M}8}}$ + 0.107 (±0.041) ${}_{{\varvec{SS}4}}^{\varvec{K}} \varvec{Tr}_{{\varvec{e} - \varvec{v} - \varvec{h}}}^{{\varvec{M}21\left( {\varvec{M}8} \right)}}$ + 0.029 (±0.012) ${}_{{\varvec{MP}4}}^{{\varvec{AC}\left[ 7 \right]\_\varvec{K}}} \varvec{Tr}_{{\varvec{a} - \varvec{e} - \varvec{c}}}^{{\varvec{M}19\left( {\varvec{M}13} \right)}}$ − 0.058 (±0.022) $\left[ {{}_{{\varvec{SS}1}}^{{\varvec{AC}\left[ 2 \right]\_\varvec{K}}} \varvec{TrQB}_{{\varvec{a} - \varvec{c}}}^{{\varvec{M}27}} } \right]^{\varvec{C}}$

^a^See Additional file 1: Table S7 for nomenclature of the QuBiLS-MIDAS descriptors

On the other hand, the Tables 7 and 8 show the comparisons with respect to other approaches reported in the literature, as well as the results obtained by the models based on *total QuBiLS*-*MIDAS MDs* exclusively (see Additional file 1: Table S4 for information related with the best models from 3 to 9 variables). In this manner, the importance of considering local-fragments (atom-types or group) in the calculation of the QuBiLS-MIDAS MDs and subsequently in the building of QSAR models can be analyzed. As can be observed in both tables, the performance of the QuBiLS-MIDAS models is superior when local-fragments are considered with respect to those QuBiLS-MIDAS models that do not use them. Particularly, it can be noted that in 6 of the 8 datasets studied the $Q_{loo}^{2}$ parameter is rather comparable, while better performances are attained according to $Q_{ext}^{2}$. Both parameters for the COX2 dataset present the best improvements, achieving in the external prediction a value greater than 49 % of the total variance, while no other QSAR procedure outperforms this threshold. On the other hand, only in the DHFR and GPB datasets does the utilization of the *local*-*fragment QuBiLS*-*MIDAS MDs* not influence the performance of the developed QSAR models. It can thus be stated that considering a mixture of *total and local*-*fragment QuBiLS*-*MIDAS MDs* in building of QSAR models contributes to the improvement of the predictive ability.

**Table 7:** Comparison of the cross-validation statistic parameter $\left( {Q_{loo}^{2} } \right)$ obtained from the QuBiLS-MIDAS models with respect to the performance achieved by 15 QSAR procedures

	ACE	ACHE	BZR	COX2	DHFR	GPB	THER	THR
QuBiLS-MIDAS^a^	*0.7756*	*0.6574*	*0.6931*	*0.6313*	*0.7055*	*0.8124*	*0.7530*	*0.8149*
QuBiLS-MIDAS^b^	*0.7713*	*0.6521*	*0.6886*	*0.6064*	*0.7055*	*0.8124*	*0.7495*	*0.8047*
CoMFA [23]	0.68	0.52	0.32	0.49	0.65	0.42	0.52	0.59
COMSIA basic [23]	0.65	0.48	0.41	0.43	0.63	0.43	0.54	0.62
COMSIA extra [23]	0.66	0.49	0.45	*0.57*	0.65	0.61	0.51	*0.72*
EVA [23]	0.70	0.42	0.40	0.45	0.64	0.58	0.48	0.47
HQSAR [23]	*0.72*	0.34	0.42	0.50	0.69	*0.66*	0.49	0.50
2D [23]	0.68	0.32	0.36	0.49	0.51	0.31	0.62	0.62
2.5D [23]	*0.72*	0.31	0.35	0.55	0.53	0.46	0.66	0.52
SAMFA-RF [43]	0.69	*0.58*	0.43	0.38	0.70	*0.66*	0.52	0.53
SAMFA-SVM [43]	0.52	0.29	0.38	0.39	0.57	0.53	0.18	0.39
SAMFA-PLS [43]	0.65	0.54	0.49	0.40	0.68	0.61	0.60	0.56
Fingerprints Library [44]	0.69	0.57	*0.56*	0.55	*0.76*	0.53	0.53	0.58
O3Q [45]	0.69	0.52	0.42	0.48	0.70	0.55	0.48	0.59
O3QMFA [46]	0.65	0.41	0.41	0.43	0.69	0.30	0.47	0.65
O3A/O3Q [45]	0.71	0.55	0.46	0.46	0.66	0.50	*0.67*	0.68
COSMO*sar3D* [46]	0.71	0.53	0.45	0.54	0.69	0.61	0.58	0.74

^a^ ${\text{Q}}_{\text{loo}}^{2}$ values corresponding to the best model reported considering total and local-fragment QuBiLS-MIDAS indices (see Table 6)

^b^ ${\text{Q}}_{\text{loo}}^{2}$ values corresponding to the best model reported considering only total QuBiLS-MIDAS indices (see Additional file 1: Table S4)

Italic values correspond to the best results reported in the literature and those obtained by the QuBiLS-MIDAS 3D-MDs

**Table 8:** Comparison of the external predictive accuracy $\left( {Q_{ext}^{2} } \right)$ attained by the QuBiLS-MIDAS models with respect to the generalization ability achieved with 12 QSAR procedures

	ACE	ACHE	BZR	COX2	DHFR	GPB	THER	THR
QuBiLS-MIDAS^a^	*0.7422*	*0.6309*	*0.5692*	*0.4932*	*0.6405*	*0.8283*	*0.7248*	*0.7674*
QuBiLS-MIDAS^b^	*0.7255*	*0.5989*	*0.5459*	*0.4660*	*0.6405*	*0.8283*	*0.7061*	*0.7498*
CoMFA [23]	0.49	0.47	0.00	0.29	0.59	0.42	0.54	0.63
COMSIA basic [23]	0.52	0.44	0.08	0.03	0.52	0.46	0.36	0.55
COMSIA extra [23]	0.49	0.44	0.12	0.37	0.53	0.59	0.53	0.63
EVA [23]	0.36	0.28	0.16	0.17	0.57	0.49	0.36	0.11
HQSAR [23]	0.30	0.37	0.17	0.27	0.63	0.58	0.53	−0.25
2D [23]	0.47	0.16	0.14	0.25	0.47	−0.06	0.14	0.04
2.5D [23]	0.51	0.16	0.20	0.27	0.49	0.04	0.07	0.28
O3Q [45]	0.69	0.67	0.17	0.32	0.60	0.50	0.51	0.67
O3QMFA [46]	0.45	0.61	0.13	0.37	0.59	0.29	0.49	0.60
O3A/O3Q [45]	0.54	0.65	0.24	0.28	0.53	0.41	−0.18	0.30
COSMO*sar3D* [46]	0.62	0.61	0.13	*0.43*	0.58	0.63	0.59	0.66
2D-FPT [47]	*0.713* ^L^	*0.714* ^N^	*0.378* ^L^	0.329^N^	*0.683* ^N^	*0.667* ^L^	*0.649* ^L^	*0.737* ^N^

^a^ ${\text{Q}}_{\text{ext}}^{2}$ values corresponding to the best model reported considering total and local-fragment QuBiLS-MIDAS indices (see Table 6)

b${\text{Q}}_{\text{ext}}^{2}$ values corresponding to the best model reported considering only total QuBiLS-MIDAS indices (see Additional file 1: Table S4)

^L^2D-FPT-based linear models

^N^2D-FPT-based non-linear models

Italic values correspond to the best results reported in the literature and those obtained by the QuBiLS-MIDAS 3D-MDs

Also, it can be observed from Table 7 that the cross-validation performances achieved by the QuBiLS-MIDAS models have comparable-to-superior behavior with respect to the approaches reported in the literature. Until now, the best $Q_{loo}^{2}$ value for the datasets ACE, ACHE, BZR, COX2, GPB, THER and THR had been attained by the procedures HQSAR (and 2.5D) [${\text{Q}}_{\text{loo}}^{2}$ = 0.72], SAMFA-RF (${\text{Q}}_{\text{loo}}^{2}$ = 0.58), All-Shortest Path [ASP] Fingerprint (${\text{Q}}_{\text{loo}}^{2}$ = 0.56), COMSIA extra (${\text{Q}}_{\text{loo}}^{2}$ = 0.57), HQSAR (and SAMFA-RF) [${\text{Q}}_{\text{loo}}^{2}$ = 0.66], O3A/O3Q (${\text{Q}}_{\text{loo}}^{2}$ = 0.67) and COMSIA extra (${\text{Q}}_{\text{loo}}^{2}$ = 0.72), respectively, by using PLS, Random Forest (RF) or Support Vector Machine (SVM) techniques. However, all these previous results are clearly outperformed by the QuBiLS-MIDAS models [(ACE, ${\text{Q}}_{\text{loo}}^{2}$ = 0.7756), (ACHE, ${\text{Q}}_{\text{loo}}^{2}$ = 0.6574), (BZR, ${\text{Q}}_{\text{loo}}^{2}$ = 0.6931), (COX2, ${\text{Q}}_{\text{loo}}^{2}$ = 0.6313), (GPB, ${\text{Q}}_{\text{loo}}^{2}$ = 0.8124), (THER, ${\text{Q}}_{\text{loo}}^{2}$ = 0.7530) and (THR, ${\text{Q}}_{\text{loo}}^{2}$ = 0.8149)], which were built with MLR that is a simpler method than those employed in the reported results. In the specific case of the DHFR dataset, although the attained value (${\text{Q}}_{\text{loo}}^{2}$ = 0.7055) with the QuBiLS-MIDAS approach is not better than the current best result (ASP fingerprint, ${\text{Q}}_{\text{loo}}^{2}$ = 0.76), the former is superior to the remaining QSAR procedures. However, it is important to remark that the best model (ASP fingerprint + SVM) for the DHFR dataset does not have the external prediction value ($Q_{ext}^{2}$) reported and thus the corresponding $Q_{loo}^{2}$ could be overoptimistic.

According to the external predictions, it can be observed in the Table 8 that the models based on QuBiLS-MIDAS indices yield comparable-to-superior performances with respect to the results reported in the literature. Specifically, the models for ACE (${\text{Q}}_{\text{ext}}^{2}$ = 0.7422), BZR (${\text{Q}}_{\text{ext}}^{2}$ = 0.5692), COX2 (${\text{Q}}_{\text{ext}}^{2}$ = 0.4932), GPB (${\text{Q}}_{\text{ext}}^{2}$ = 0.8283), THER (${\text{Q}}_{\text{ext}}^{2}$ = 0.7248) and THR (${\text{Q}}_{\text{ext}}^{2}$ = 0.7674) test sets outperform the best results reported up to date for each dataset previously mentioned, which correspond to COSMO*sar3D* (${\text{Q}}_{\text{ext}}^{2}$ = 0.43) in COX2 and to the 2D-FPT methodology in the other datasets [(ACE, ${\text{Q}}_{\text{ext}}^{2}$ = 0.713), (BZR, ${\text{Q}}_{\text{ext}}^{2}$ = 0.378), (GPB, ${\text{Q}}_{\text{ext}}^{2}$ = 0.667), (THER, ${\text{Q}}_{\text{ext}}^{2}$ = 0.649) and (THR, ${\text{Q}}_{\text{ext}}^{2}$ = 0.737)]. The 2D-FPT models were developed by using SQS framework that determines linear and non-linear models (see Table 8), while the model corresponding to COSMO*sar3D* is based on the PLS technique. Even so, the obtained MLR models have better predictive accuracy, even when these are compared with respect to more complex or similar procedures.

As for the ACHE and DHFR datasets, the predictive power obtained for models built with the QuBiLS-MIDAS approach is inferior to the best results reported so far in the literature. In the former dataset, the methods 2D-FPT (${\text{Q}}_{\text{ext}}^{2}$ = 0.714), O3Q (${\text{Q}}_{\text{ext}}^{2}$ = 0.67) and O3A/O3Q (${\text{Q}}_{\text{ext}}^{2}$ = 0.65) offer better predictions than the proposed model (${\text{Q}}_{\text{ext}}^{2}$ = 0.6309), albeit this can be considered as suitable (explains 63 % of total variance). Additionally, when the DHFR test set is taken into account the 2D-FPT approach (${\text{Q}}_{\text{ext}}^{2}$ = 0.683) has more predictive ability than the corresponding QuBiLS-MIDAS model (${\text{Q}}_{\text{ext}}^{2}$ = 0.6405), but the latter is superior to the remaining methodologies. Nonetheless, it is important to highlight that the procedures O3Q and O3A/O3Q are alignment dependent and thus their use is generally restricted to congeneric datasets [45]. In the specific case of the 2D-FPT methodology for ACHE and DHFR datasets, the achieved results are based on non-linear models while the proposed outcomes are determined with linear models.

The obtained results evidence that the QuBiLS-MIDAS MDs properly codify structural information of the molecules considering interactions among *N* (*N* = 2, 3, 4) atoms and thus are suitable for developing QSAR models that contribute to the prediction of biological activity in novel structures. However, notwithstanding the comparable-to-superior predictions achieved by the proposed models, it is important to statistically validate these results.

### Statistical analysis of the external predictive accuracy

**Fig. 1:**
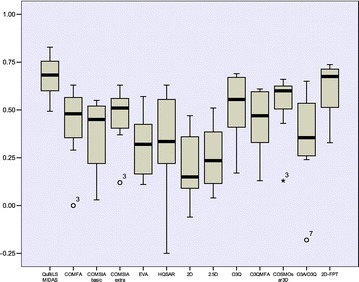
Boxplot graphic for the external predictive accuracy achieved by each QSAR methodology considered in this manuscript

To perform this analysis the values corresponding to the external predictions (${\text{Q}}_{\text{ext}}^{2}$) obtained by the QuBiLS-MIDAS models were taken into consideration as well as the ones reported in the literature over the external compounds belonging to each dataset (see Table 8). Firstly, a descriptive analysis through boxplot graphics was performed (with SPSS software) and the obtained results are represented in Fig. 1. As can be observed, the QuBiLS-MIDAS and 2D-FTP models tend to have a similar behavior and superior to the remaining procedures. Also, it can be noted that the highest prediction among all procedures analyzed is achieved by the QuBiLS-MIDAS models. In addition, taking into account the graphics corresponding to the QuBiLS-MIDAS and 2D-FPT approaches, it can be concluded that the predictions obtained by the former are less scattered than those attained by the latter and thus, the QuBiLS-MIDAS models have a more suitable external predictive ability irrespective of the chemical dataset analyzed. However, these results are not enough to state that the models based on QuBiLS-MIDAS MDs are statistically the best.

Therefore, an exploratory study was performed to analyze the normality of the data by using Kolmogorov–Smirnov (K–S) test corrected by Lilliefors [53] and the Shapiro–Wilk test [54]. This was done in order to guarantee that the variable ${\text{Q}}_{\text{ext}}^{2}$ is not normally distributed, at least for one model, and so to ensure that the non-parametric tests are the proper choice. As can be observed in Additional file 1: Table S5, the *null hypotheses of normality* can only be rejected with a high certainty for ${\text{Q}}_{\text{ext}}^{2}$ values in the 2D-FTP and COSMO*sar3D* models, although with Shapiro–Wilk test the rejection of the null hypothesis is achieved for COMSIA basic as well. Therefore the non-parametric tests may be considered as suitable for this statistical analysis.

Subsequently, a Friedman test [55] for multiple comparisons was performed taking into consideration the results of all QSAR procedures. As can be seen in Additional file 1: Table S6A, there are global differences among the considered methods, with the QuBiLS-MIDAS models being those with the best performance followed by the 2D-FPT, O3Q and COSMO*sar3D* approaches, respectively, with a Kendall’s W [56] concordance level of 0.607 (see Additional file 1: Table S6B). In order to determine the specific statistical differences a Wilcoxon signed-ranks test [57] was carried out (with R software) by using Benjamini and Hochberg [58] (BH) as the adjustment method (one-tailed *p values* calculation) for controlling the *false discovery rate* (FDR). The results of this analysis are shown in Table 9, where a significant *pvalue* (*p* value <0.05) means that the row approach is superior to the corresponding column. So, it can be noted that the QuBiLS-MIDAS models yield statistically better predictions than the other methodologies considered, with the exception of the 2D-FPT approach.

**Table 9:** Wilcoxon signed-rank test for pairwise multiple hypothesis tests by using BH as adjustment method for controlling FDR. It shows the one-tailed *p*-*values* for the greater alternative

	2D	2.5D	EVA	COMSIA basic	HQSAR	O3QMFA	CoMFA	O3A/O3Q	COMSIA extra	COSMO sar3D	O3Q	2D-FPT
2.5D	0.115	–	–	–	–	–	–	–	–	–	–	–
EVA	0.138	0.402	–	–	–	–	–	–	–	–	–	–
COMSIA basic	0.137	0.115	0.323	–	–	–	–	–	–	–	–	–
HQSAR	0.203	0.380	0.197	0.402	–	–	–	–	–	–	–	–
O3QMFA	0.046	0.046	0.138	0.241	0.312	–	–	–	–	–	–	–
CoMFA	0.051	0.089	0.115	0.241	0.367	0.703	–	–	–	–	–	–
O3A/O3Q	0.089	0.089	0.277	0.556	0.402	0.654	0.727	–	–	–	–	–
COMSIA extra	0.031	0.051	0.045	0.051	0.164	0.427	0.249	0.272	–	–	–	–
COSMOsar3D	0.027	0.022	0.036	0.022	0.051	0.054	0.027	0.068	0.015	–	–	–
O3Q	0.015	0.022	0.022	0.015	0.186	0.051	0.042	0.051	0.203	0.698	–	–
2D-FPT	0.015	0.015	0.015	0.015	0.015	0.022	0.015	0.015	0.022	0.068	0.015	–
QuBiLS MIDAS	*0.015*	*0.015*	*0.015*	*0.015*	*0.015*	*0.015*	*0.015*	*0.022*	*0.015*	*0.015*	*0.022*	*0.138*

Italic values indicate statistically significant differences of the QuBiLS-MIDAS models with respect to the other QSAR methodologies

### Analysis of the predictive ability according to conformer generation methods

The conformer generation constitutes an important step when chemoinformatics tasks are performed, particularly in the computer-aided drug design, where the outcomes of a virtual screening process may depend on 3D structures employed to build the procedure to be used, e.g. a QSAR model [59]. Therefore, in this section an evaluation of the sensibility of the QuBiLS-MIDAS MDs to the different conformer generation methods is performed in order to comprehend how these could affect in the performance of the indices. To this end, the software FROG2 [60], RDKit [61], BALLOON [62], OpenBabel [63] and Standardizer ChemAxon [64] were employed to generate the 3D structures, taking as starting point the SMILES representations corresponding to the eight compound datasets considered in this report.

**Fig. 2:**
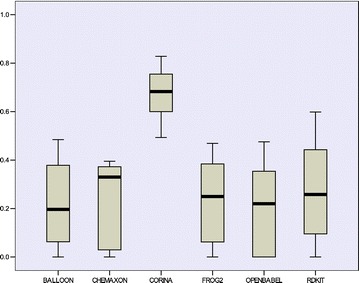
Boxplot graphic for the external predictive accuracy achieved by the QSAR models reported in this manuscript (see Table 6) and fitted using structures generated by CORINA software, over the corresponding test sets optimized by five different toolkits

Firstly, a study with the purpose of knowing if the models developed using the training structures generated with CORINA (see Table 6) are applicable to the test structures generated with the previously mentioned programs was performed. The external predictive abilities obtained after performing this study are graphically represented in Fig. 2. These results are significantly inferior to those achieved with the test sets based on CORINA (see Additional file 1: Table S8), with the exception of RDKIT. This demonstrates that QSAR models based on QuBiLS-MIDAS MDs are not suitable to predict biological activity into compounds optimized with other procedure different from than used for the training structures. Thus, it can be stated that the performance of the QuBiLS-MIDAS MDs depend on 3D conformations from which are computed.

It is important to highlight that the previous results do not mean that CORINA software is the most suitable to generate the 3D structures to be used in the development of the QSAR models based on QuBiLS-MIDAS MDs. In this sense, in order to prove this assertion the following simple workflow was carried out considering the conformations generated by each previously mentioned program (including CORINA) for each chemical dataset:

- 8640 *two*-*linear algebraic indices* (Additional file 1: Table S9) were computed.
- *CfsSubsetEval* feature selection procedure, implemented in WEKA software, was applied in order to retain those MDs with high correlation according to dependent-variable and with low intercorrelation among them.
- The MLR-GA procedure implemented in MobyDigs software was employed to build 9-variable models performing 100,000 iterations and considering the tabu list options of removing MDs with correlation equal or greater than 0.95, fourth order moment greater than 8 and standardized entropy lesser than 0.3. The fitness function used was the statistical parameter $Q_{loo}^{2}$.
- The model with the highest $Q_{loo}^{2}$ value was selected as the best model, to which the external predictive ability was determined.

Table 10 shows the external predictive power of the models developed from different 3D conformations, as well as the average of the rankings corresponding to the conformer generation methods considered in this study. As can be observed, the best predictions are achieved by the models built from 3D molecular structures generated by FROG2 procedure, followed by the results obtained from the methods CORINA, CHEMAXON, RDKIT, OPENBABEL and BALLOON, respectively. However, in Additional file 1: Table S10 is demonstrated through a Friedman test that there exists no global statistic differences among previous predictions, which proves, at least for this preliminary study that with QuBiLS-MIDAS MDs can be developed QSAR models with good predictive accuracy irrespective of the procedure used to obtain optimized structures.

**Table 10:** External predictive accuracy achieved by QSAR models developed from 3D molecular structures generated with six different programs

	ACE	ACHE	BZR	COX2	DHFR	GPB	THER	THR	Rank average
BALLOON	0.3296	0.1943	0.3949	0.2451	0.3758	0.0000	0.0000	0.0000	4.5
CHEMAXON	0.5504	0.1343	0.4163	0.3361	0.2978	0.1687	0.0000	0.1386	3.375
CORINA	0.4133	0.0556	0.3628	0.2865	0.4288	0.2767	0.1915	0.2334	3.25
FROG2	0.4832	0.3535	0.3635	0.3393	0.3786	0.2712	0.3264	0.1457	2.125
OPENBABEL	0.3993	0.1306	0.1715	0.2775	0.3460	0.4742	0.2806	0.0803	4
RDKIT	0.4181	0.1770	0.3024	0.2189	0.5008	0.4511	0.0000	0.0710	3.75

Note that for the forthcoming version of QuBiLS-MIDAS software, RDKIT program will be incorporated in the QuBiLS-MIDAS software as a built-in option for conformer generation. This is due to the fact that FROG2 procedure can only be accessed using a web browser, while CORINA and CHEMAXON software are not freely available for use. In addition, according to a study performed in Ref. [65] in order to assess the quality of the conformations generated by several free methods, RDKIT tends to generate the most similar conformations to the experimental structures, in addition to being the second fastest among all toolkits analyzed.

## Conclusions

In this report the predictive accuracy of the novel alignment-free geometric molecular descriptors based on N-linear algebraic maps (so called QuBiLS-MIDAS) has been examined. To this end, QSAR models for predicting the biological activity in eight molecular datasets were developed by using MLR as statistical technique. The results obtained with the QuBiLS-MIDAS models were compared with respect to several QSAR procedures reported in the literature according to the correlation coefficients achieved with the *leave*-*one*-*out cross*-*validation*$\left( {Q_{loo}^{2} } \right)$ and *external prediction*$\left( {Q_{ext}^{2} } \right)$ methods, and generally superior performances were observed with this QuBiLS-MIDAS framework.

A few exceptions were observed: for the $Q_{loo}^{2}$ parameter, the QuBiLS-MIDAS approach is exclusively outperformed by the ASP-based (fingerprint) method in the DHFR dataset, while for the $Q_{ext}^{2}$ parameter, the QuBiLS-MIDAS method yields inferior results with respect to the 2D-FPT methodology in the DHFR and ACHE test set, respectively. Also, inferior $Q_{ext}^{2}$ values are yielded by the QuBiLS-MIDAS approach with respect to the O3Q and O3A/O3Q procedures in the ACHE test set. However, these previous methodologies are based on techniques more complex than MLR and/or cannot be used in non-congeneric datasets because are alignment-depend. Thus, considering the maximum parsimony principle (Ockham’s razor), the QuBiLS-MIDAS approach seems to be more suitable than the other QSAR methods.

Additionally, several steps for statistically validating the obtained results are detailed. In this sense, the external predictive ability of the developed models was compared with respect to other methodologies by means of the multiple comparison tests. It was demonstrated that the QuBiLS-MIDAS models yield the best predictions, and that these are significantly superior in 11 of the 12 methodologies compared. Therefore, it can be suggested that the 3D Algebraic N-linear molecular descriptors (also known as QuBiLS-MIDAS) are suitable for extracting structural information of the molecules and thus, constitute a promissory alternative to build models that contribute to the prediction of pharmacokinetic, pharmacodynamics and toxicological properties of novel compounds.

## Additional file

10.1186/s13321-016-0122-x The statistical parameters and equations of the 3–9 variable QSAR models developed and the corresponding outputs for the statistical analysis performed.
